# Possible Paraneoplastic Amyotrophic Lateral Sclerosis Presenting as Respiratory Failure in a Breast Cancer Patient: A Case Report

**DOI:** 10.1002/ccr3.73447

**Published:** 2026-08-31

**Authors:** Maryam Albaji, Khashayar Ashkboos, Kosar Bagherzadeh

**Affiliations:** ^1^ Department of Pulmonary Disease Sina Hospital, Tehran University of Medical Sciences Tehran Iran; ^2^ Tehran University of Medical Sciences Tehran Iran; ^3^ Zanjan University of Medical Sciences Zanjan Iran

**Keywords:** amyotrophic lateral sclerosis, breast neoplasms, paraneoplastic syndromes, respiratory insufficiency, seronegativity

## Abstract

A 63‐year‐old female with metastatic breast cancer presented with acute hypercapnic respiratory failure secondary to severe diaphragmatic dysfunction and mixed upper and lower motor neuron signs. Serum onconeural and neural surface antibody panels were negative, and initial cerebrospinal fluid analysis revealed albuminocytologic dissociation. Immunotherapy with intravenous immunoglobulin (IVIG) produced no clinical improvement, necessitating invasive mechanical ventilation and gastrostomy. Applying the 2021 PNS‐Care consensus criteria, the case fulfills criteria for possible paraneoplastic amyotrophic lateral sclerosis (ALS), although coincidental sporadic disease cannot be excluded. A markedly elevated erythrocyte sedimentation rate (ESR) was observed, largely attributable to skeletal metastases and localized pulmonary collapse. Clinicians must maintain a high index of suspicion for motor neuron disease in cancer patients with unexplained hypoventilation.

## Key Clinical Message

In patients with active cancer and unexplained hypercapnic respiratory failure, motor neuron disease should be evaluated. In seronegative presentations, marked systemic inflammatory markers often reflect metastatic burden or secondary pulmonary collapse rather than a specific neurodegenerative biomarker in paraneoplastic syndromes.

## Graphical abstract

In a breast cancer patient with acute hypercapnic respiratory failure, paraneoplastic ALS was diagnosed despite negative onconeural antibodies. Red flags included diaphragmatic paralysis, ESR of 120 mm/h, and CSF protein of 246 mg/dL. Clinicians should consider this diagnosis even when antibody panels are negative.

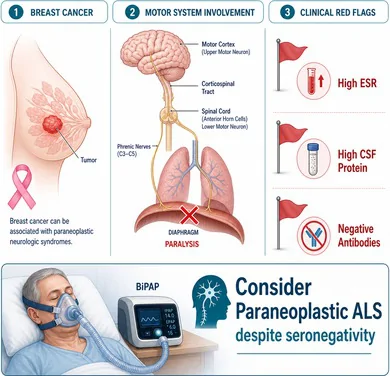

## 1 Introduction

Amyotrophic lateral sclerosis (ALS) is a fatal neurodegenerative disorder characterized by progressive degeneration of upper motor neurons (UMN) in the motor cortex and lower motor neurons (LMN) in the brainstem and spinal cord [1, 2]. While ALS remains a relatively rare disease, with an estimated global incidence between 0.26 and 23.46 per 100,000 person‐years, it represents the most prevalent adult‐onset motor neuron disease [3]. The clinical course is marked by relentless neuromuscular weakness and muscular atrophy, leading to a median survival time of only 2 to 5 years from symptom onset [4]. Respiratory failure resulting from diaphragmatic and intercostal muscle denervation represents the primary cause of mortality in the vast majority of cases [5, 6].

Paraneoplastic neurological syndromes (PNS) constitute a spectrum of immune‐mediated complications triggered by an antitumor immune response that aberrantly cross‐reacts with neural antigens [7, 8]. Under the 2021 updated PNS‐Care consensus criteria, neurological phenotypes are categorized by their risk association into “high‐risk” (classical) or “intermediate‐risk” (non‐classical) syndromes [7]. Motor neuron disease (ALS phenotype) is recognized as an intermediate‐risk paraneoplastic phenotype [7, 9]. Motor neuron disease has been reported in association with small cell lung cancer, lymphoma, and breast adenocarcinoma [10, 11, 12]. Distinguishing paraneoplastic motor neuron disease from idiopathic sporadic ALS is clinically crucial, as paraneoplastic variants may occasionally stabilize following successful antitumor therapy or immunotherapy, whereas idiopathic ALS is inexorably progressive [10, 11].

Diagnosing paraneoplastic ALS is particularly challenging when acute hypercapnic respiratory failure is the initial presentation—a rare onset seen in fewer than 3% of ALS patients, frequently leading to misdiagnosis of primary cardiopulmonary disorders [5, 13]. Diagnostic difficulty is further compounded in seronegative presentations, where classical onconeural antibodies are absent [7, 14]. Here, we report a complex case of a 63‐year‐old female with pre‐existing metastatic breast carcinoma presenting with acute hypercapnic respiratory failure as the initial manifestation of an ALS phenotype meeting criteria for possible paraneoplastic ALS.

## 2 Case History/Examination

A 63‐year‐old female presented to our emergency department with progressive dyspnea and generalized body weakness that had begun 15 days prior. Before admission to our tertiary center, she had been evaluated at another facility for suspected pulmonary thromboembolism (PTE); following negative diagnostic testing, she had been discharged with a presumed psychosomatic disorder. Despite generalized weakness, she remained able to perform basic activities of daily living until two days before presentation. She denied fever, chills, cough, sputum production, orthopnea, or focal chest pain.

Her medical history was significant for Type 2 diabetes mellitus and invasive breast adenocarcinoma diagnosed 9 months earlier, with documented osteolytic metastases to the cervical (C5–C6), thoracic (T7–T12), and lumbar (L1–L5) spine. She had undergone partial mastectomy, adjuvant chemotherapy, and completed 30 sessions of radiotherapy 2 months prior to this presentation. She had also been hospitalized for severe COVID‐19 pneumonia 6 months earlier. She was a non‐smoker with passive smoke exposure.

Upon admission, the patient was alert and oriented but markedly tachypneic with shallow breathing. Pulmonary auscultation revealed diminished breath sounds bilaterally without wheezing or crackles. Her oxygen saturation was 89% on ambient air. Neurological examination showed symmetric limb strength of 4/5 proximally and distally, which appeared disproportionately preserved relative to the severity of her acute respiratory compromise.

## 3 Differential Diagnosis, Investigations and Treatment

### 3.1 Investigations and Initial Laboratory Findings

Arterial/venous blood gas analysis revealed acute‐on‐chronic respiratory acidosis (pH: 7.34, pCO2: 60 mmHg, HCO3: 30.4 mEq/L). Routine hematological and biochemical panels, including serum urea (35 mg/dL), creatinine (0.7 mg/dL), and C‐reactive protein (CRP: 4.2 mg/L), were within normal ranges. However, the erythrocyte sedimentation rate (ESR) was markedly elevated at 120 mm/h. While extreme ESR elevation is atypical for pure neurodegenerative conditions, in this clinical context it was recognized as secondary to her extensive skeletal metastases (C5–L5), tissue hypoxemia, and localized pulmonary atelectasis rather than a specific neurodegenerative biomarker.

Additional investigations demonstrated elevated serum Vitamin B12 (1577 pg/mL), low folic acid (1.3 ng/mL), elevated homocysteine (19.5 μmol/L), and elevated serum LDH (480 U/L). Initial laboratory data are summarized in Table 1.

**TABLE 1:** Initial laboratory findings upon admission.

Parameter	Value	Reference range
Venous blood gas (VBG)		
pH	7.34	7.35–7.45
pCO_2_	60 mmHg	35–45 mmHg
HCO_3−_	30.4 mEq/L	22–26 mEq/L
Complete blood count		
WBC	7900 /μL	4000–11,000 /μL
Hemoglobin	12.4 g/dL	12.0–16.0 g/dL
Platelets	249,000 /μL	150,000–450,000 /μL
Biochemistry and inflammatory		
Urea	35 mg/dL	15–45 mg/dL
Creatinine	0.7 mg/dL	0.6–1.1 mg/dL
Sodium	137 mmol/L	135–145 mmol/L
Potassium	3.9 mmol/L	3.5–5.1 mmol/L
AST	22 U/L	7–56 U/L
ALT	20 U/L	15–45 U/L
CRP	4.2 mg/L	< 5.0 mg/L
PCT	0.6 ng/mL	< 0.5 ng/mL
ESR	120 mm/h	< 20 mm/h
D‐Dimer	Negative	Negative
Metabolic and vitamins		
Vitamin B_12_	1577 pg/mL	200–900 pg/mL
Folic Acid	1.3 ng/mL	> 3.0 ng/mL
Homocysteine	19.5 μmol/L	5.0–15.0 μmol/L
Serum LDH	480 U/L	140–280 U/L

**FIGURE 1:**
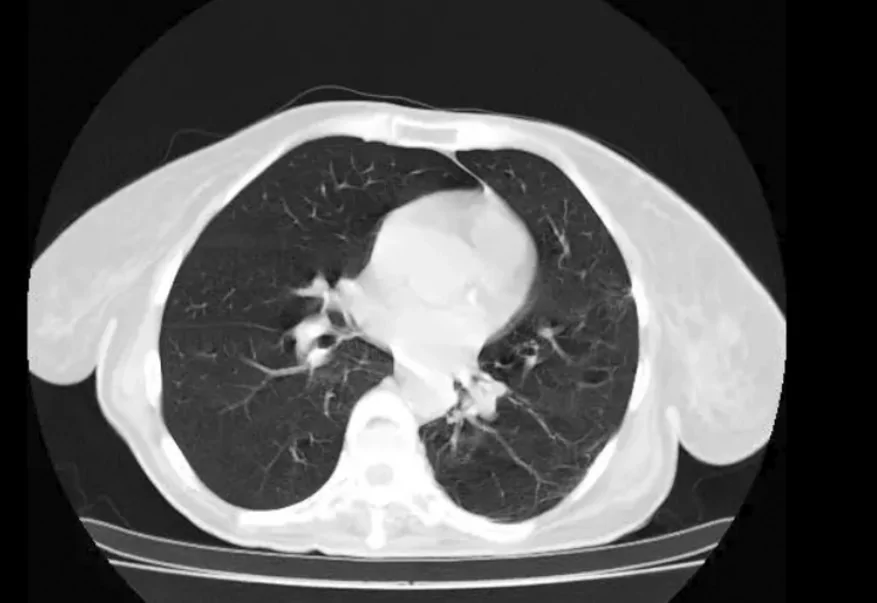
Chest CT scan. The chest CT image demonstrates clear lung fields with no evidence of consolidation, pleural effusion, atelectasis, or pulmonary embolism. The absence of significant pulmonary pathology supports a neuromuscular or central cause for the patient's hypoventilation.

A non‐contrast chest CT scan demonstrated clear lung parenchyma without focal consolidation, pleural effusion, pneumothorax, or large‐airway obstruction that could explain the severe hypoventilation (Figure 1).

### 3.2 Hospital Course and Specialty Consultations

The patient was initially treated with bronchodilators and respiratory physiotherapy; however, her hypercapnia worsened. During hospitalization, she experienced an acute decrease in consciousness and apnea as her pCO2 reached 101 mmHg, necessitating emergency endotracheal intubation and mechanical ventilation.

An initial neurological evaluation revealed symmetric limb strength of 4/5 with an asymmetric extensor plantar response (positive Babinski on the left and flexor on the right). Following stabilization and reduction in pCO2, she was successfully extubated and transitioned to non‐invasive ventilation (NIV). While on NIV, a comprehensive neurological re‐examination noted generalized hyperreflexia (3+ deep tendon reflexes), widespread fasciculations across limbs and shoulder girdle, and normal cranial nerve function without ocular or sensory involvement.

Advanced diagnostic investigations were performed:

- Electromyography and Nerve Conduction Velocity (EMG‐NCV): Showed widespread active denervation (fibrillation potentials, positive sharp waves) and chronic neurogenic changes (large‐amplitude, polyphasic motor unit action potentials) across cervical, thoracic, and lumbosacral segments, along with mixed UMN signs (hyperreflexia, extensor plantar response), consistent with motor neuron disease.
- Diaphragmatic Ultrasonography: Confirmed severe reduction in right hemidiaphragmatic excursion (< 10 mm) and absent thickening during inspiration, establishing severe diaphragmatic paresis.
- Neuroimaging: Brain MRI revealed mild diffuse cortical atrophy and chronic microvascular white matter changes without intracranial metastases. Cervical spine CT revealed degenerative changes and known stable osteolytic lesions without acute neural compression.
- Cerebrospinal Fluid (CSF) Analysis:
  1. *First LP*: Demonstrated albuminocytologic dissociation with elevated CSF protein of 246 mg/dL (Reference: 15–45 mg/dL), glucose of 75 mg/dL, and CSF LDH of 270 U/L, with 0 WBCs.
  2. *Second LP* (*performed 10 days later*): Showed acellular fluid (0 WBCs, 60 RBCs), normal glucose (65 mg/dL), and a subnormal protein level (6 mg/dL). CSF cytology was negative for malignant cells. PCR assays for Tuberculosis and *Brucella*, fungal/bacterial cultures, and CSF ADA (4.2 U/L) were negative.
- Paraneoplastic and Autoimmune Antibody Panels: Comprehensive serum antibody testing for classical onconeural and neuronal surface antibodies (including Anti‐Hu, Yo, Ri, CV2, Amphiphysin, Ma2/Ta, SOX1, GAD65, VGKC, NMDA‐R, AMPA‐R, CASPR2, LGI1), as well as anti‐AChR (0.3 nmol/L) and anti‐MuSK antibodies, was completely negative (Table 2).

**TABLE 2:** Paraneoplastic antibody panel results.

Immunology test	Result	Reference interval
Acetylcholine receptor Ab	0.3 nmol/L	< 0.40 – negative > = 0.40–< 0.50—borderline > = 0.50—positive
Paraneoplastic neurologic syndromes		Negative: 0–5 Borderline: 6–10 Positive: > 11
Amphiphysin	Negative	
CV2	Negative	
PNMA2 (Ma2/Ta)	Negative	
Ri	Negative	
Yo	Negative	
Hu	Negative	
Recoverin	Negative	
SOX1	Negative	
Titin	Negative	
Zic4	Negative	
GAD65	Negative	
Tr(DNER)	Negative	
Control	Negative	
Anti Musk Ab	Negative (titer)	Negative < 1/20 Positive = > 1/20
Anti NMDA‐R	Negative	
AMPAR1 and AMPAR2	Negative	
GABARB1/B2	Negative	
CASPR2	Negative	
LGI 1	Negative	
Anti VGKC	Negative	

*Note:* Results of the paraneoplastic neurological syndrome panel. All tested onconeural antibodies (including Anti‐Hu, Yo, Ri, and CV2) were negative, indicating a seronegative status.

## 4 Conclusion and Results (Outcome and Follow‐Up)

Following diagnostic evaluation, the patient developed progressive dysphagia and severe impairment of secretion clearance. Recurrent hypercapnic respiratory decompensation necessitated re‐intubation.

Based on the progressive motor neuron syndrome in the setting of active metastatic malignancy and CSF albuminocytologic dissociation, a diagnosis of possible paraneoplastic ALS was established. She received a 5‐day course of intravenous immunoglobulin (IVIG, 0.4 g/kg/day); however, no objective clinical or electrophysiological improvement occurred.

To facilitate long‐term airway management and enteral nutrition, an elective surgical tracheostomy was performed, and a percutaneous endoscopic gastrostomy (PEG) tube was placed. The patient was discharged in a stable but ventilator‐dependent state to a specialized home‐care setting with portable mechanical ventilatory support.

## 5 Discussion

We have described a complex case of a 63‐year‐old female with pre‐existing metastatic breast carcinoma presenting with acute hypercapnic respiratory failure as the initial manifestation of an ALS phenotype. In oncology patients presenting with rapid motor neuron signs, distinguishing between a true paraneoplastic syndrome and coincidental sporadic ALS remains an important diagnostic challenge [10, 11].

### 5.1 Diagnostic Classification: Applying the 2021 PNS‐Care Criteria

Motor neuron disease is classified as an intermediate‐risk (“non‐classical”) phenotype for paraneoplastic neurological syndromes [7]. According to the updated 2021 PNS‐Care consensus criteria [7], a “Definite” PNS diagnosis requires high‐risk onconeural antibodies. In our patient, a comprehensive screening panel covering both intracellular onconeural and cell‐surface antigens was entirely negative.

Under the standardized PNS‐Care scoring algorithm:

- Intermediate‐risk phenotype (Motor Neuron Disease): 1 point
- Active cancer diagnosed within 2 years of neurological onset: 2 points
- Seronegativity: 0 points
- Lack of sustained improvement following immunotherapy: 0 points

With a cumulative score of 3 points, this case fulfills the diagnostic criteria for “Possible PNS” [7]. While the absence of antibodies precludes a definitive diagnosis, approximately 15% to 20% of clinically validated paraneoplastic syndromes are seronegative [8, 14]. In these cases, the pathogenic process is predominantly mediated by CD8+ cytotoxic T‐cell‐driven neuronal destruction rather than antibody‐mediated humoral injury [14].

### 5.2 Inflammatory Markers and Coincidental Malignancy

Classic sporadic ALS is a non‐inflammatory neurodegenerative disease characterized by normal systemic inflammatory markers. While earlier clinical series by Forsyth et al. [15] suggested that extreme ESR elevation in motor neuron syndromes warrants an immediate search for underlying malignancy, inflammatory markers in our patient must be interpreted with caution. The markedly elevated ESR (120 mm/h) in this case was largely secondary to widespread skeletal metastases (C5–L5), tissue hypoxemia, and localized pulmonary atelectasis rather than a specific central neuroinflammatory marker.

Large register‐based epidemiological studies by Fang et al. [16] and Freedman et al. [17] have shown that while cancer survivors may have modest alterations in neurological disease incidence, the co‐occurrence of ALS and common malignancies such as breast cancer is often coincidental due to overlapping peak demographic distributions (ages 60–75). Furthermore, Rojas‐Marcos et al. [18] observed that motor neuron presentations in breast and gynecologic malignancies frequently pursue a relentless course, highlighting the difficulty in establishing causality without autopsy or tissue‐level immunopathology.

### 5.3 Cerebrospinal Fluid Findings

Initial lumbar puncture revealed marked albuminocytologic dissociation (protein: 246 mg/dL). While mild protein elevations occur in up to 30% of sporadic ALS cases, protein levels exceeding 150 mg/dL are rare [19]. Severe CSF hyperproteinorrhachia occurs in nearly two‐thirds of paraneoplastic neurological disorders, reflecting increased blood–brain barrier permeability or inflammatory radiculopathy [19]. The marked reduction in protein (6 mg/dL) on repeat lumbar puncture 10 days later likely represents technical sampling variability, localized dilutional artifact, or transient disruption of the blood‐spinal cord barrier during acute hypercapnic stress.

### 5.4 The Respiratory‐Onset Phenotype

Acute respiratory failure as the initial manifestation of motor neuron disease is exceedingly rare, occurring in fewer than 3% of ALS presentations [5, 6, 20]. Chen et al. [20] and Oh et al. [21] highlighted that when diaphragmatic denervation disproportionately precedes limb weakness, diagnostic delay is frequent, and patients are routinely misdiagnosed with primary cardiopulmonary disorders. In our patient, bedside diaphragmatic ultrasound provided decisive objective evidence of bilateral diaphragmatic dysfunction.

### 5.5 Therapeutic Implications and Limitations

Unlike classic sporadic ALS, paraneoplastic motor neuron syndromes have occasionally been reported to stabilize or improve following tumor resection or immunotherapy [10, 11]. In our patient, high‐dose IVIG did not yield clinical or electrophysiological recovery. This lack of response is consistent with the irreversible loss of anterior horn cells and phrenic motor neurons prior to treatment initiation, as well as the relative resistance of cytotoxic T‐cell‐mediated paraneoplastic neurodegeneration to humoral‐targeted immunotherapies like IVIG [10, 14].

### 5.6 Limitations

A definitive causal paraneoplastic link cannot be established with absolute certainty due to seronegativity, lack of response to immunotherapy, and the absence of post‐mortem histopathology. Coincidental sporadic respiratory‐onset ALS in a patient with metastatic breast carcinoma remains an important alternative possibility.

## Author Contributions

**Maryam Albaji:** conceptualization, investigation, writing – review and editing, visualization, methodology, supervision, data curation, project administration. **Khashayar Ashkboos:** investigation, writing – original draft, writing – review and editing, visualization, methodology. **Kosar Bagherzadeh:** investigation, writing – review and editing, validation.

## Funding

The authors have nothing to report.

## Ethics Statement

Ethical approval was not required for this case report in accordance with the institutional policy of Tehran University of Medical Sciences.

## Consent

Written informed consent was obtained from the patient to publish this case report and any accompanying images.

## Conflicts of Interest

The authors declare no conflicts of interest.

## Data Availability

Data sharing is not applicable to this article as no datasets were generated or analyzed during the current case report.
